# Application of cardiovascular interventions to decrease blood loss during hepatectomy: a systematic review and meta-analysis

**DOI:** 10.1186/s12871-023-02042-y

**Published:** 2023-03-22

**Authors:** Hui Ye, Hanghang Wu, Bin Li, Pengfei Zuo, Chaobo Chen

**Affiliations:** 1 Department of Anesthesiology, ZhongDa Hospital, Southeast University, No. 87 Dingjiaqiao, Gulou District, Nanjing, Jiangsu Province 210009 China; 2 Department of Immunology, Ophthalmology and ENT, School of Medicine, Complutense University, Madrid, Spain; 3 Department of Cardiology, ZhongDa Hospital, Southeast University, Nanjing, China; 4 Department of General Surgery, Xishan People’s Hospital of Wuxi city, No. 1128 Dacheng Road, Xishan District, Wuxi, 214105 China; 5 Department of Hepatic-Biliary-Pancreatic Surgery, The Affiliated Drum Tower Hospital of Nanjing University Medical School, Nanjing, China

**Keywords:** Cardiovascular interventions, Blood loss, Transfusion, Hepatectomy, Meta-analysis

## Abstract

**Background:**

Perioperative bleeding and allogeneic blood transfusion are generally thought to affect the outcomes of patients. This meta-analysis aimed to determine the benefits and risks of several cardiovascular interventions in patients undergoing hepatectomy.

**Methods:**

In this systematic review and meta-analysis, randomised controlled trials (RCTs) were searched in the Cochrane Library, Medline, Embase, and Web of Science to February 02, 2023. RCTs focused on cardiovascular interventions aimed at reducing blood loss or blood transfusion requirements during hepatectomy were included. The primary outcomes were perioperative blood loss amount, number of patients requiring allogeneic blood transfusion and overall occurrence of postoperative complications. The secondary outcomes were operating time, perioperative mortality rate, postoperative liver and kidney function and length of hospital stay.

**Results:**

Seventeen RCTs were included in the analysis. A total of 841 patients who underwent hepatectomy in 10 trials were included in the comparative analysis between low central venous pressure (CVP) and control groups. The forest plots showed a low operative bleeding volume [(mean difference (MD): -409.75 mL, 95% confidence intervals (CI) -616.56 to -202.94, *P* < 0.001], reduced blood transfusion rate [risk ratio (RR): 0.47, 95% CI 0.34 to 0.65, *P* < 0.001], shortened operating time (MD: -13.42 min, 95% CI -22.59 to -4.26, *P* = 0.004), and fewer postoperative complications (RR: 0.76, 95% CI 0.58 to 0.99, *P* = 0.04) in the low CVP group than in the control group. Five and two trials compared the following interventions, respectively: ‘acute normovolaemic haemodilution (ANH) vs control’ and ‘autologous blood donation vs control’. ANH and autologous blood donation could not reduce the blood loss amount but greatly decreased the number of patients requiring allogeneic blood transfusion. No benefits were found in the rate of mortality and length of postoperative hospital stay in any of the comparisons.

**Conclusion:**

Lowering the CVP seems to be effective and safe in adult patients undergoing hepatectomy. ANH and autologous blood donation should be used as a part of blood management for suitable patients in certain circumstances.

**Trial registration:**

PROSPERO, CRD42022314061.

**Supplementary Information:**

The online version contains supplementary material available at 10.1186/s12871-023-02042-y.

## Background

Hepatectomy is a complex procedure for the treatment of liver tumours. Perioperative management remains a challenge for both anaesthesiologists and surgeons owing to the high risk of perioperative morbidity and mortality [1]. The 30-day mortality rate is 1.9% for partial hepatectomy and 5.8% for extensive hepatectomy [2].

Blood loss and blood transfusion requirements during hepatectomy are generally thought to affect the outcomes of patients [3]. Recently, enhanced recovery after surgery (ERAS) has gained considerable acceptance worldwide and is beneficial in shortening the length of hospital stay and reducing the rate of morbidity [4]. Hence, an effective method to reduce bleeding is urgently needed to avoid complications and promote rapid recovery after hepatectomy.

Many methods have been attempted, including improvements in surgical modalities, application of various pharmacological agents, occlusion of blood flow to the liver, and cardiovascular interventions [5–7]. Cardiovascular interventions, including lowering the central venous pressure (LCVP) [8], dilution of blood [7], and donation of autologous blood [6], are widely applied to reduce blood loss during hepatectomy. LCVP (central venous pressure [CVP] reduction to < 5 mmHg) to reduce intraoperative bleeding has become a routine practice during liver surgery [9]. In acute normovolaemic haemodilution (ANH), anaesthesiologists withdraw 200 or 400 ml whole blood from patients in the operating room and simultaneously replace it with crystalloid or colloid solution [10]; in autologous blood donation, patients’ blood is stored several weeks prior to surgery [11]. The withdrawn blood can be reinfused during or after surgery, if needed, which can reduce the use of allogeneic blood, thereby decreasing the incidence of postoperative complications and promoting patient recovery after major liver surgery [12].

All these efforts, including LCVP, ANH and autologous blood donation, aim to reduce intra- and postoperative bleeding and allogeneic blood transfusion; however, the safety and efficacy in hepatectomy remain controversial. The majority of centres still utilise intravenous fluid restriction and vasodilation to decrease the CVP, potentially leading to systemic hypovolaemia. Therefore, there are concerns regarding the possibility of impaired kidney function, poor tissue oxygenation, and haemodynamic instability. On the other hand, with widespread health measures, rigorous pre-donor screening, and donor blood testing, the risk of transfusion-transmitted infectious disease is extremely low. Simultaneously, the risks associated with autologous blood transfusions have increased. In addition, high costs markedly reduce the application of autologous blood transfusion.

In general, a comprehensive assessment of the benefits and risks of treatments would be helpful for perioperative management of patients undergoing hepatectomy. Some systematic reviews published previously have reported that LCVP during hepatectomy can reduce operative blood loss. However, no cardiopulmonary interventions seemed to decrease perioperative morbidity or offer any long-term survival benefit [13–15]. Furthermore, the trials included in these studies had a high risk of bias, which limits the representativeness of their results. Several studies that sought new methods to reduce the CVP have been published thereafter [16–20]. In some trials, two or more methods were applied together, which needs to be considered in the analysis [16, 21]. Therefore, an updated and comprehensive meta-analysis is needed to further assess the clinical benefits and risks of these interventions in patients undergoing hepatectomy.

## Materials and methods

This meta-analysis followed Preferred Reporting Items for Systematic Reviews and Meta-Analyses (PRISMA) guidelines [22, 23]. The registration number of the study in PROSPERO is CRD42022314061.

### Search strategy

We searched the Cochrane Library, Medline, Embase, and Web of Science databases up to February 02, 2023 to identify original studies focused on reducing blood loss in patients undergoing hepatectomy. The search strategy for all databases is presented in the Additional file 1. In addition, the references of the articles were manually searched to identify any potentially relevant trials.

### Selection criteria

The selection criteria were as follows: (1) Types of studies: all randomised clinical trials (RCTs); (2) Types of participants: patients undergoing hepatectomy regardless of aetiology, being major or minor liver resections, normal or cirrhotic liver; (3) Types of interventions: any cardiovascular intervention aimed at reducing blood loss or blood transfusion requirements during hepatectomy, such as LCVP, ANH, autologous blood donation, and hypotension control, compared with control conditions (no intervention or other techniques) were included. Co-interventions were allowed when they were performed simultaneously in the trial groups. (4) Types of outcome measures: intraoperative blood loss and transfusion requirements as outcomes. Reviews, letters, case reports, ongoing trials or trials on animals, and repeated or overlapping previous literature were excluded.

### Data collection and extraction

Two investigators independently reviewed all the titles and abstracts of the articles to determine their eligibility. The relevant full-text articles were carefully reviewed to assess whether they met the inclusion criteria. Inter-researcher disagreements were resolved by a third investigator. The following data were independently extracted from the eligible studies by the two investigators: 1. year and language of publication; 2. country in which the trial was conducted; 3. year the trial took place; 4. inclusion and exclusion criteria; 5. number of major hepatectomies; 6. number of patients with cirrhosis; 7. number of intervention and control; 8. outcomes; and 9. risk of bias. When the data were not presented in a form that facilitated data synthesis, the authors were contacted using published communication tools. When the authors did not respond, the medians and ranges were converted to means ± standard deviations (SDs) using the methods described by McGrath et al [24].

### Risk of bias assessment

In this systematic review, the risk of bias was evaluated using the Revised Cochrane risk-of-bias tool for randomized trials (RoB 2). Two authors independently assessed the risk of bias of the included trials following the instructions in the Cochrane Handbook for Systematic Reviews of Interventions [25]. The following risk of bias components were judged from each trial: random sequence generation, allocation concealment, blinding of participants and personnel, blinding of outcome assessment, incomplete outcome data, selective reporting, and other biases.

Quality of evidence was determined using the Grading of Recommendations Assessment, Development, and Evaluation (GRADE) system for outcomes based on the following criteria: study design, risk of bias, inconsistency, indirectness, imprecision and others. The quality of evidence was graded as high, moderate, low and very low.

### Outcomes

The main outcomes in this meta-analysis were as follows: 1. perioperative blood loss amount; 2. number of patients requiring allogeneic blood transfusion and overall mean number of units or volume of allogeneic whole blood transfused; and 3. overall occurrence of postoperative complications.

Additional outcomes were as follows: 1. operating time; 2. perioperative mortality rate; 3. postoperative liver function indicators [alanine transaminase (ALT), aspartate aminotransferase (AST), and total bilirubin (TB) levels]; 4. postoperative kidney function indicators [blood urea nitrogen (BUN) and serum creatinine (Cr) levels]; and 5. length of hospital stay.

### Data analysis

Statistical analysis was conducted using the Cochrane Review Manager (version: 5.4.1; The Nordic Cochrane Centre) and Stata MP (version 16.0; StataCorp LP, USA). Dichotomous outcomes, including the postoperative complication rate, intraoperative blood transfusion requirement, and mortality rate, were presented as pooled risk ratios (RRs) and 95% confidence intervals (CIs). When only a trial was included in the comparative analysis, Fisher’s exact test was performed using Stata MP. Meanwhile, continuous outcomes, including the blood loss amount, operating time, and hospital stay length, were presented as mean differences (MDs) with 95% CIs. Statistical significance was set at *P* < 0.05. When the values reported in the original articles were provided as medians and interquartile (IQR) ranges, and we could not retrieve the mean ± SD values from the authors, we used statistical methods to convert the values [26].

Heterogeneity was assessed using the *I*^2^ statistic and adjusted as low, medium, and high when the *I*^2^ values were 25%, 50%, and 75%, respectively. When there was significant heterogeneity, we used the random-effects model. Otherwise, the fixed-effects model was used.

A subgroup analysis was performed according to the technique of CVP reduction for all CVP-lowering interventions, while no subgroup analysis was conducted for ANH and autologous blood donation because of the few trials included in this review. To further assess the stability of the primary outcomes, we performed a sensitivity analysis to identify influential cases for the meta-analysis. In addition, funnel plots were generated to examine potential publication bias.

## Results

### Included trials

**Fig. 1:**
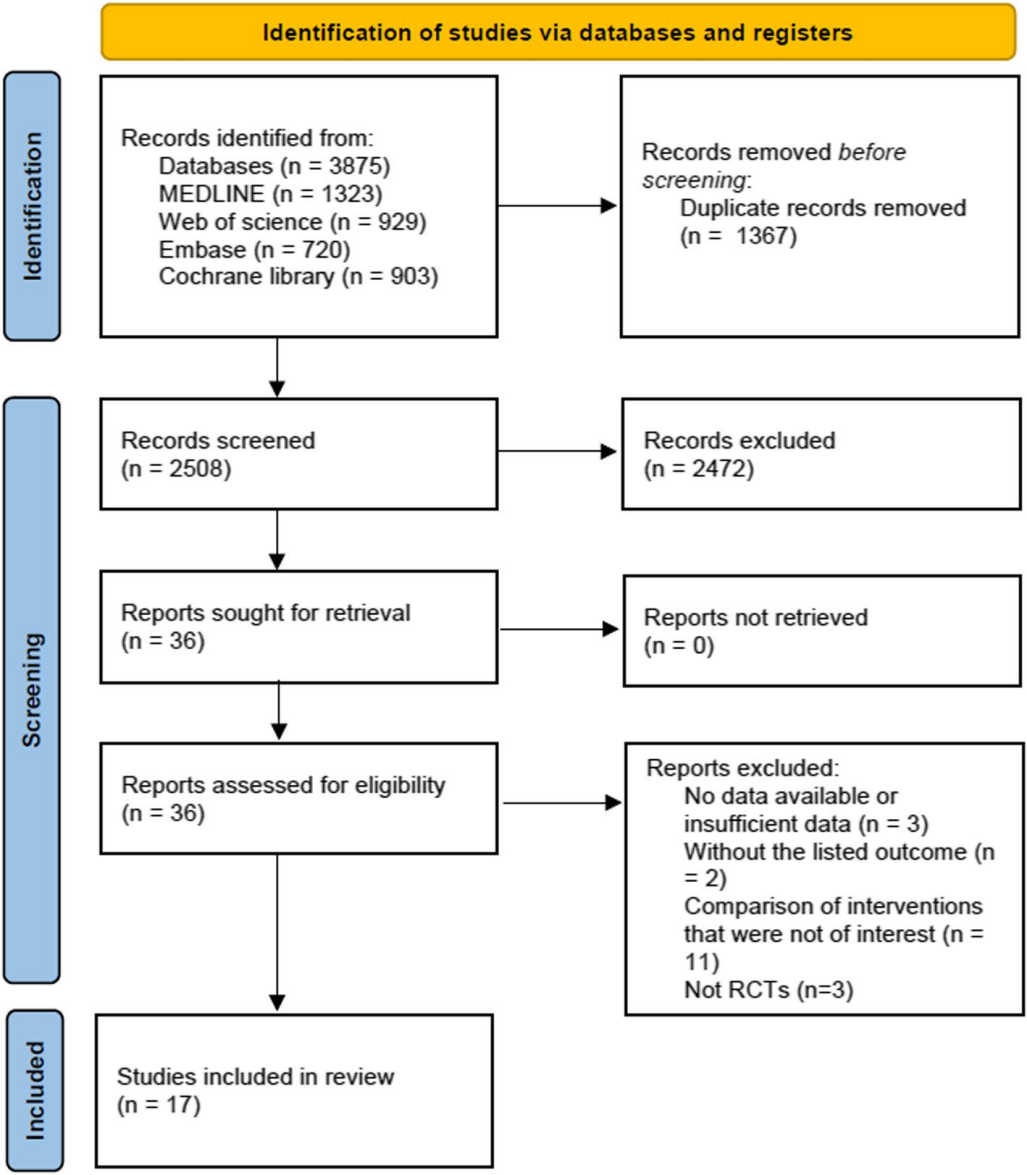
Literature search and study selection processes according to the Preferred Reporting Items for Systematic Reviews and Meta-Analyses (PRISMA) guidelines

A total of 3678 articles were identified through the manual comprehensive search in the databases. No additional records were identified by scanning the reference lists of the related articles. 17 RCTs met the inclusion criteria and were included in the analysis [6–8, 16–21, 27–34]. Figure 1 illustrates the process of study selection according to the PRISMA 2020 guidelines [35].

### Study characteristics

Details regarding the patients and interventions in the included trials are shown in Table 1.

**Table 1:** Summary of the details of the included studies

**Study**	**Country**	**Sample size**	**Men/Women**	**Average age**	**Indication**	**NO. of cirrhotics**	**Type of surgery**	**NO. of major hepatectomies**	**Intervention**	**Comparison**
**Chen H (1997) **[27]	USA	I:7 C:9	7/9	57.8 years	MCC	1	Major hepatic resection	16	ANH	Control
**Eid E (2005) **[28]	Eygpt	I:15 C:15	15/15	Not stated	Not stated	Not stated	Consecutive hepatectomy	Not stated	Limiting infusion volume, nitroglycerine, fentanyl to low CVP	Routine fluid management
**El-kharboutly (2004) **[29]	Eygpt	I:20 C:20	23/17	51.1 years	Not stated	40	Elective liver resection	25	Low CVP using nitroglycerine infusion	Control
**Guo JR (2010) **[30]	China	I:15 C:15	22/8	65 years	Not stated	Not stated	Hepatic carcinectomy	Not stated	Acute normovolemic dilution	No intervention
**Guo JR (2015) **[16]	China	I:40 C:20	38/22	50 years	Not stated	Not stated	Liver resection	Not stated	1. Low CVP 2. Low CVP + Acute normovolemic dilution	Control
**Hashimoto (2007) **[31]	Japan	I:40 C:39	49/30	33.5 years	Liver donor	0	Liver graft procurement	77	Autologous blood donation	Control
**Jarnagin WR (2008) **[7]	USA	I:63 C:67	69/61	53.5 years	HCC; MCC; GBC; MLT	71	Major hepatic resection	130	Acute normovolemic dilution	Control
**Junrungsee S (2021) **[17]	Thailand	I:59 C:61	74/46	56.6 years	HCC; ICC; GBC; MCC	83	Elective hepatectomy	None reported	Intrahepatic inferior vena cava clamping to low CVP	No IVC clamping
**Kajikawa (1994) **[6]	Japan	I:21 C:21	Not stated	Not stated	HCC	42	Liver resection	12	Autologous blood donation	Control
**Kato M (2008) **[32]	Japan	I:43 C:42	Not stated	66 years	PLC; GBC; ICC; MLT	Not stated	Liver resection	Not stated	Clamping the intrahepatic inferior vena cava	No IVC clamping
**Matot I (2002) **[33]	Israel	I:39 C:39	31/47	56.5 years	PLC; MLT	Not stated	Elective major hepatic resection (hepatectomy or extended hepatectomy)	78	Acute normovolemic dilution	Control
**Pan YX (2020) **[20]	China	I:73 C:73	125/21	54.5 years	HCC	59	Laparoscopic hepatectomy	Not stated	Intravenous use of vasoactive drugs to low CVP	Routine management
**Ueno M (2017) **[19]	Japan	I:45 C:45	65/25	70 years	HCC; GBC; MLT; ICC	Not stated	Liver resection	32	Clamping intrahepatic inferior vena cava to maintain the CVP ≤ 3 mmHg	Control
**Wang WD (2006) **[8]	China	I:25 C:25	40/10	45.7 years	HCC	29	Hepatectomy	17	Trendelenburg’s posture, nitroglycerine, isoflurane, limiting the volume and speed of infusion to low CVP	Control
**Yao XH (2006) **[21]	China	I:20 C:10	16/14	Not stated	PLC	Not stated	Hepatic resection	Not stated	1. ANH with hypotension 2. ANH without hypotension	Control
**Yu L (2020) **[18]	China	I:70 C:69	116/33	55.1 years	PLC; MLT	37	Elective partial hepatectomy	56	Administration of nitroglycerin and esmolol using an infusion pump to maintain a low CVP	Control
**Zhow YM (2016) **[34]	China	I:50 C:51	94/7	54 years	HCC	25	Major right hepatic resection	101	Intrahepatic inferior vena cava clamping	Control

*Abbreviations: HCC* Hepatocellular carcinoma, *PLC* Primary liver carcinoma, *MLT* Metastatic liver tumor, *ICC* Intrahepatic cholangiocellular carcinoma, *GBC* Gallbladder carcinoma, *MCC* Metastasis of colorectal carcinoma

A total of 841 patients who underwent hepatectomy in 10 trials [8, 16–20, 28, 29, 32, 34] were included in the comparative analysis between low CVP (*n* = 420) and control (*n* = 421) groups. Among these 10 studies, four [17, 19, 32, 34] utilised clamping of the intrahepatic inferior vena cava (IVC) to achieve a controlled low CVP, while the remaining studies [8, 16, 18, 20, 28, 29] used other approaches, such as limiting the infusion volume, adopting the Trendelenburg position, and using vasodilators. The CVP was < 5 mmHg in the low CVP group (Ueno et al. even limited the CVP to < 3 mmHg) and > 5 mmHg in the control group [8, 16–20, 28, 29, 32, 34].

A total of 274 patients from five studies were randomised to the following comparison groups: ‘haemodilution (*n* = 134) vs control (*n* = 140)’ [7, 21, 27, 30, 33]. A total of 121 patients undergoing hepatectomy from two trials were randomised to the following comparison groups: ‘autologous blood donation (*n* = 61) vs control (*n* = 60)’ [6, 31]. Two trials [21, 30] applied two interventions simultaneously: low CVP with ANH and ANH with hypotension control. The details of these two studies have been previously described.

**Fig. 2:**
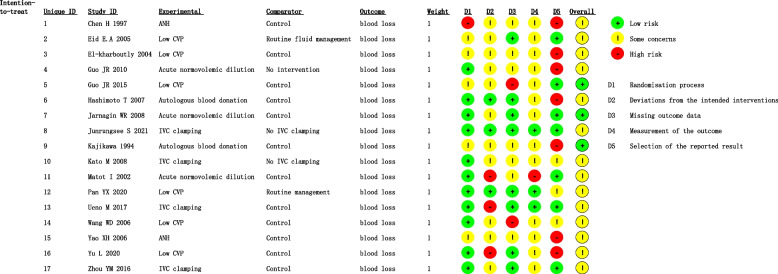
Risk of bias of the included studies

All patients in the included trials were adults. Most of the included studies were single-centre studies conducted in seven different countries. The sample sizes of the included trials varied greatly from 6 to 70. The details of the risk of bias are summarised in Fig. 2.

### Primary outcomes

#### Blood loss amount

**Fig. 3:**
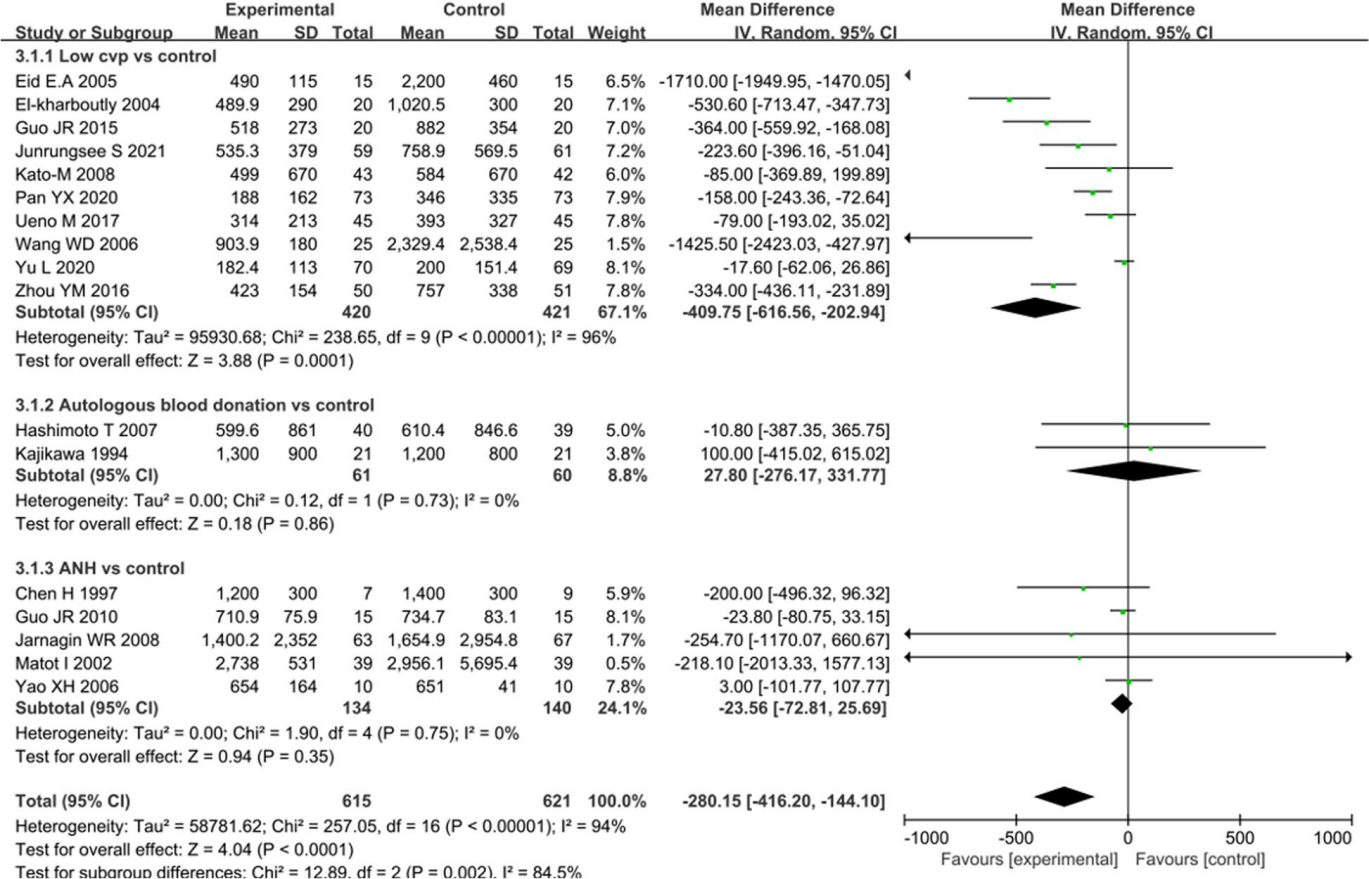
Forest plot of the meta-analysis for the intraoperative blood loss amount

All trials for each intervention reported the blood loss amount, and the details are shown in Fig. 3. Ten trials utilised CVP-lowering interventions. The operative bleeding volume was significantly lower in the low CVP group than in the control group (MD: -409.75 mL, 95% CI -616.56 to -202.94, *P* < 0.001) [8, 16–20, 28, 29, 32, 34]. However, the heterogeneity for this result was high (*I*^2^ = 96%, *P* < 0.001). Five [7, 21, 27, 30, 33] and two trials [6, 31] compared the following interventions, respectively: ‘ANH vs control’ and ‘autologous blood donation vs control’. There were no significant differences in the intraoperative blood loss amount between the groups (MD: -23.56 mL, 95% CI -72.81 to 25.69, *P* = 0.35 and MD: 27.80 mL, 95% CI -276.17 to 331.77, *P* = 0.86). One trial [21] applied a CVP-lowering treatment and hypotension control simultaneously, and another trial [30] used both CVP-lowering treatment and ANH. The blood loss amount evidently decreased in the groups that simultaneously utilised two interventions compared to the control group.

#### Blood transfusion requirements

**Fig. 4:**
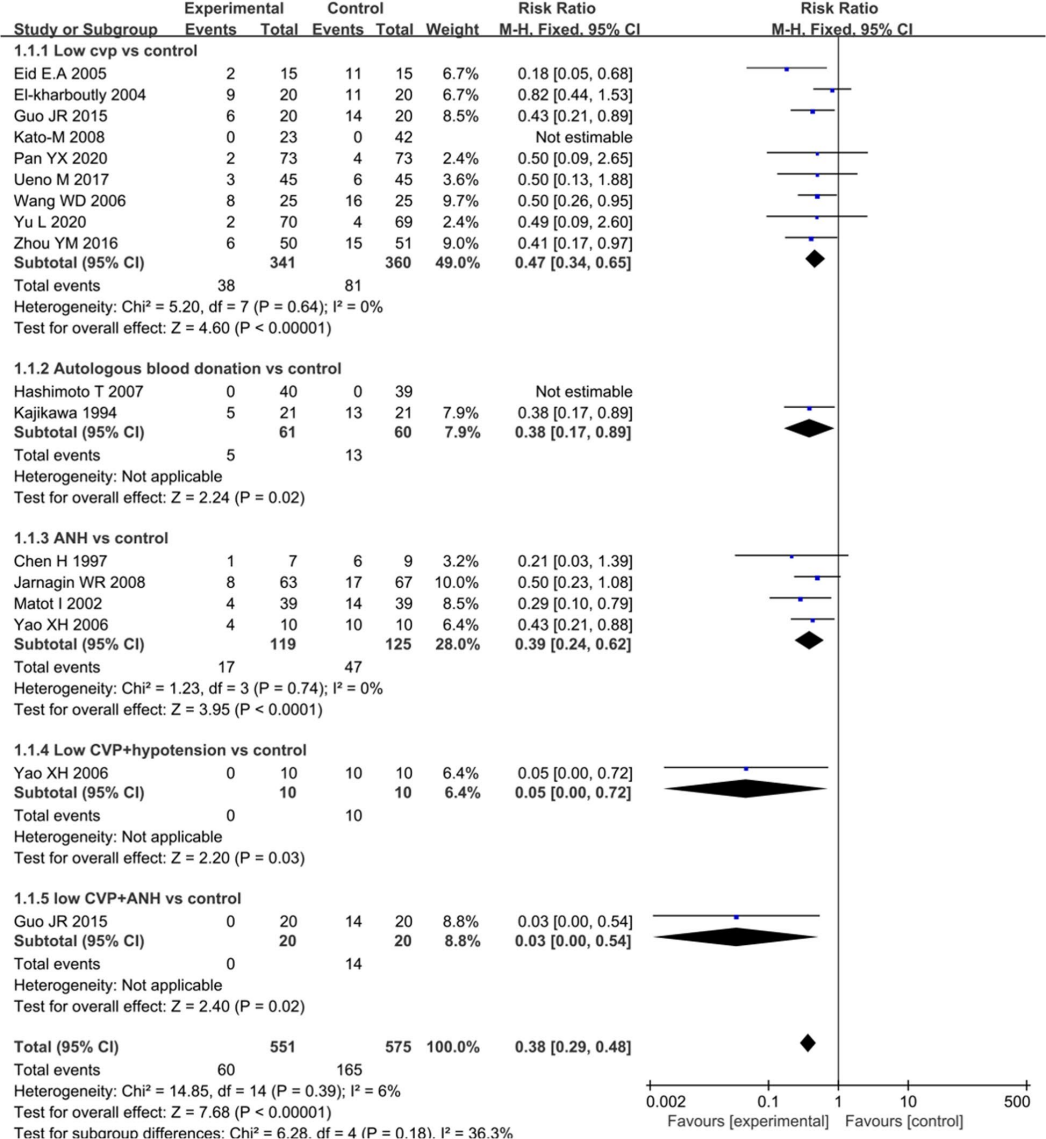
Forest plot of the meta-analysis for the number of patients requiring blood transfusion

**Fig. 5:**
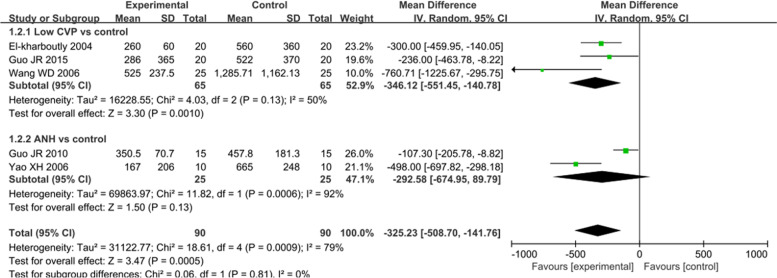
Forest plot of the meta-analysis for the total volume of blood transfused

Blood transfusion requirements were also assessed as a primary outcome of this review (Fig. 4). Nine trials compared the low CVP group with the control group. Approximately 11.1% of the patients in the low CVP group and 22.5% of those in the control group required blood transfusion (RR: 0.47, 95% CI 0.34 to 0.65, *P* < 0.001) [8, 16, 18–20, 28, 29, 32, 34] (Fig. 4). In addition, the total blood transfusion volume significantly decreased in the low CVP group compared with that in the control group (MD: -346.12 mL, 95% CI -551.45 to -140.78, *P* = 0.001) [8, 29, 30] (Fig. 5). In four trials, the number of patients requiring allogeneic blood transfusion decreased among the patients who received ANH compared with that among the controls (RR: 0.39, 95% CI 0.24 to 0.62, *P* < 0.001) [7, 21, 27, 33] (Fig. 4). However, there were no differences in the total volume of allogeneic blood transfused (MD: -292.58 mL, 95% CI -674.95 to 89.79, *P* = 0.13) [21, 30] (Fig. 5). Two trials compared autologous blood donation with control treatment, showing less requirement of blood transfusion in the intervention group than in the control group (RR: 0.38, 95% CI 0.17 to 0.89, *P* = 0.02) [6, 31] (Fig. 4). Similarly, the two trials that simultaneously applied two methods showed a significant decrease in the number of patients requiring blood transfusion in the intervention group compared with that in the control group [21, 30] (Fig. 4). Information on the components transfused (red blood cells and frozen plasma) was insufficient for analysis.

#### Postoperative complications

**Fig. 6:**
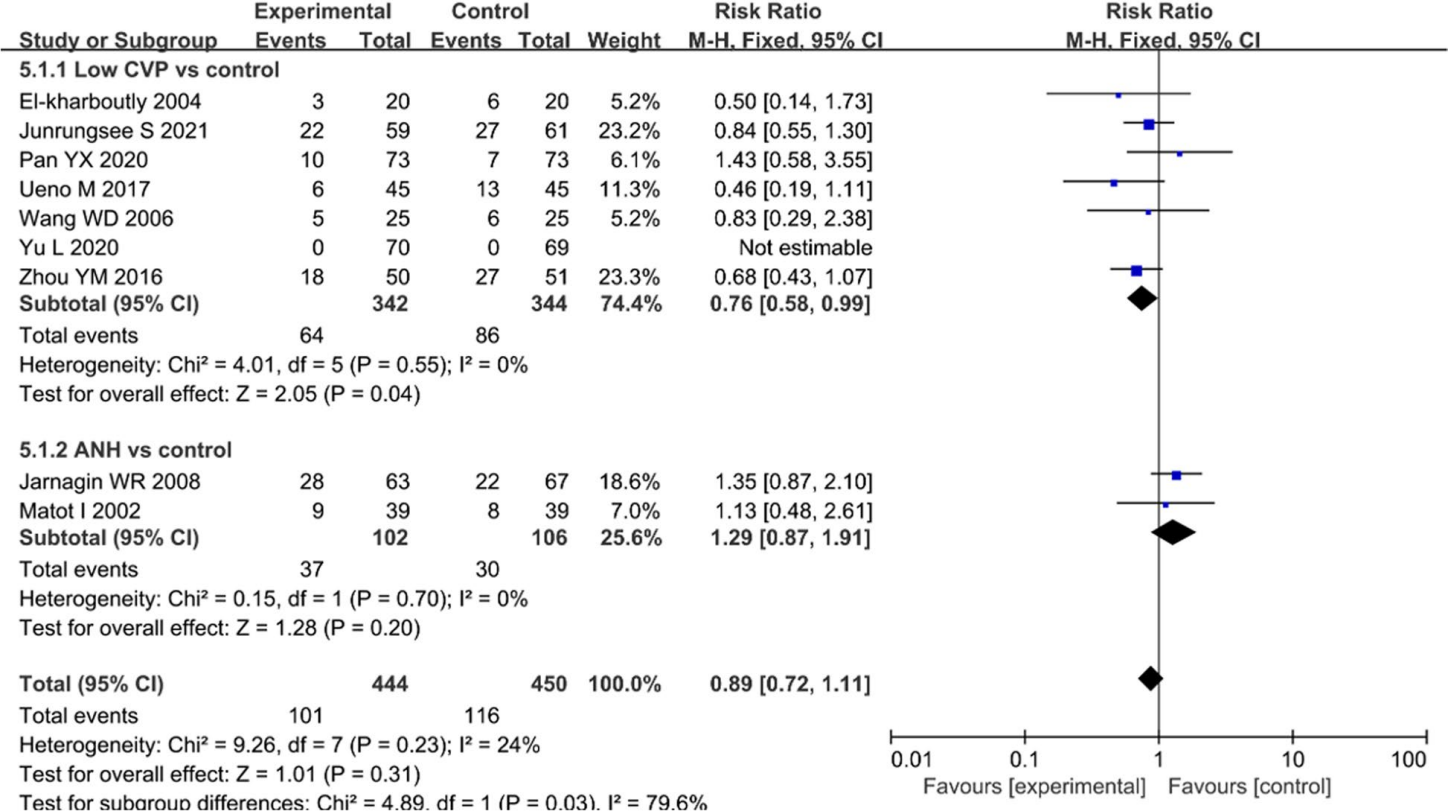
Forest plot of the meta-analysis for the postoperative complications

The low CVP group developed fewer postoperative complications than did the control group (seven trials, 64/342 vs 86/344; RR: 0.76, 95% CI 0.58 to 0.99, *P* = 0.04) [8, 17–20, 29, 34]. ANH showed no benefit in reducing the rate of postoperative adverse events compared with the control treatment (RR: 1.29, 95% CI 0.87 to 1.91, *P* = 0.20) [7, 33] (Fig. 6). Additionally, a meta-analysis was performed for four typical complications (wound infection, biliary leak, pneumonia, and intestinal obstruction), which revealed no significant differences among the variables (Supplementary Fig. 1).

#### Subgroup analysis

A subgroup analysis was performed in the low CVP category according to the method of CVP reduction (IVC clamping vs anaesthetic technique) to explore the source of high heterogeneity. The forest plots showed that IVC clamping yielded a smaller blood loss amount, less requirement for blood transfusion, and reduced complication rate than did non-IVC clamping [17, 19, 32, 34]. Meanwhile, the forest plots demonstrated that the anaesthetic technique yielded a smaller blood loss amount and less requirement for blood transfusion in the low CVP group than in the control group. However, the rate of morbidity was similar between the two groups [8, 16, 18, 20, 28, 29] (Table 2).

**Table 2:** Results of the subgroup analyses

	**MD (mL)/RR**	**95**% **CI**	***I***^***2***^	***P* value**
**Blood loss**				
IVC clamping	-193.46	-339.85 to -47.07	74%	0.01
Anesthetic technique	-607.78	-962.25 to-253.31	98%	< 0.001
**Number of patients requiring transfusion**				
IVC clamping	0.43	0.21 to 0.89	0%	0.02
Anesthetic technique	0.48	0.34 to 0.69	0%	< 0.001
**Complications**				
IVC clamping	0.70	0.52 to 0.94	0%	0.02
Anesthetic technique	0.95	0.53 to 1.71	0%	0.86

### Secondary outcomes

#### Operating time

The operating time was reported in 8 out of 10 trials, with 726 patients included in the comparative analysis between the low CVP and control groups. LCVP massively shortened the operating time compared with the control intervention (MD: -13.42 min, 95% CI -22.59 to -4.26, *P* = 0.004) [8, 16–20, 29, 34]. The combination of low CVP and ANH yielded the same effect [16]. No evident difference was observed in the comparisons between autologous blood donation and control [6, 31] and between ANH and control [27, 33] (Supplementary Fig. 2).

#### Mortality rate

No significant differences were found in the mortality rate in any of the following comparisons: low CVP vs control (six trials, low CVP: 1/265 vs control: 2/266) [8, 17, 20, 29, 32, 34] and ANH vs control (two trials, ANH: 1/73 vs control: 3/77) [7, 21]. No mortality was observed in the comparison between autologous blood donation and control [31] and between low CVP with hypotension and control [21] (Supplementary Fig. 3).

#### Postoperative liver and kidney function indicators

The postoperative liver and kidney functions were also monitored in this meta-analysis. The ALT, AST, and TB levels increased shortly after surgery and then gradually decreased 1 week after hepatectomy. Transient increases in the kidney function indicators were also observed after surgery, but which likely had a limited clinical impact. Furthermore, the forest plots showed no significant difference in the ALT, AST, TB, BUN, and Cr levels after surgery between the intervention and control groups, apart from the relatively decreased BUN and Cr levels on postoperative days 7 and 3 in the intervention group, respectively (Supplementary Figs. 4–8).

#### Postoperative hospital stay length

There was no significant difference in the length of postoperative hospital stay between the groups. Three trials (356 patients) compared low CVP with the control treatment [17, 19, 20], and only one trial compared autologous blood donation with the control treatment [31] (Supplementary Fig. 9).

#### Publication bias and sensitivity analyses

**Fig. 7:**
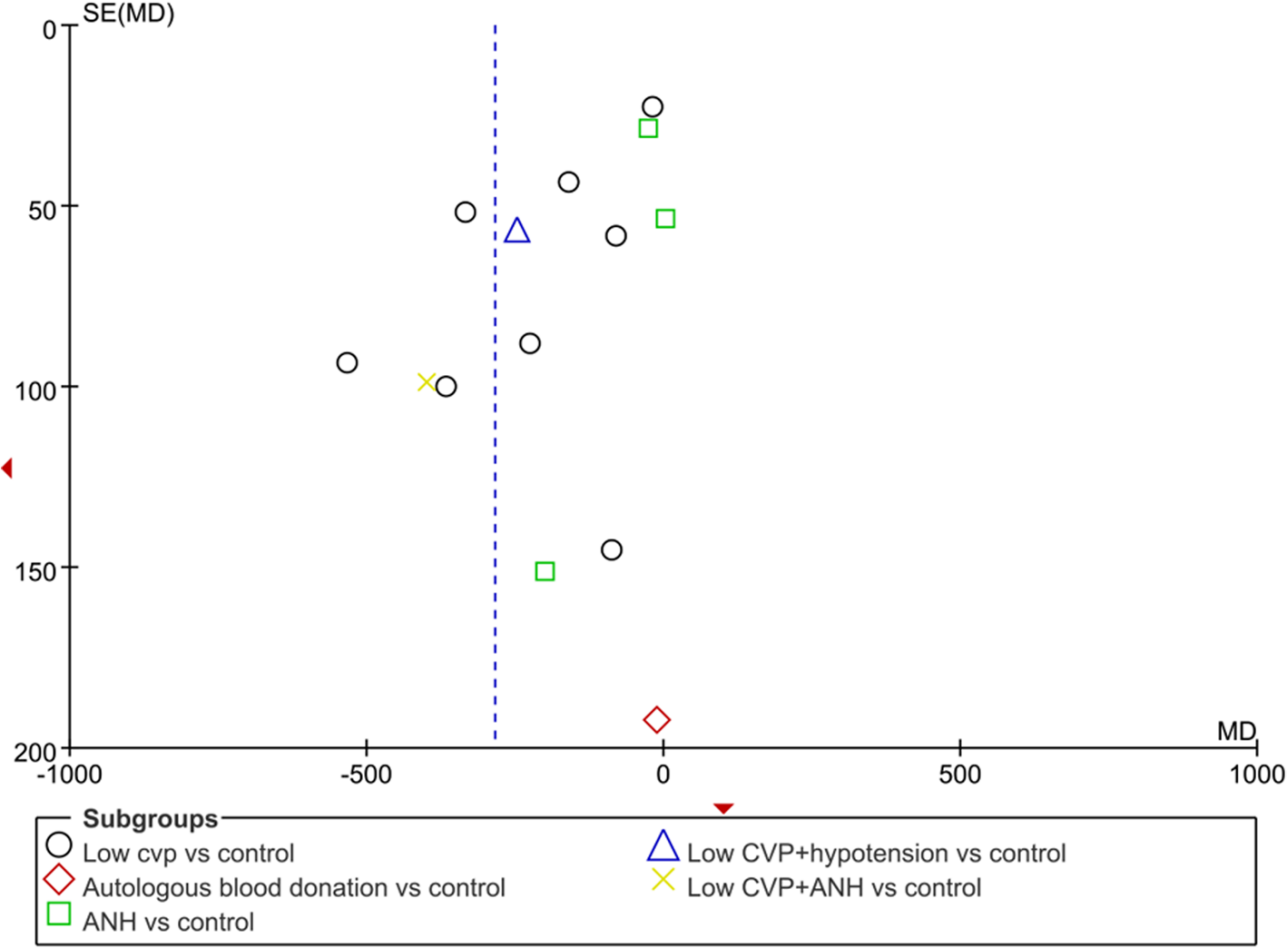
Funnel plot of the blood loss amount

Publication bias and sensitivity analyses were performed to assess blood loss. The funnel plot for the blood loss amount showed an essentially symmetrical distribution (Fig. 7), indicating no obvious publication bias.

**Fig. 8:**
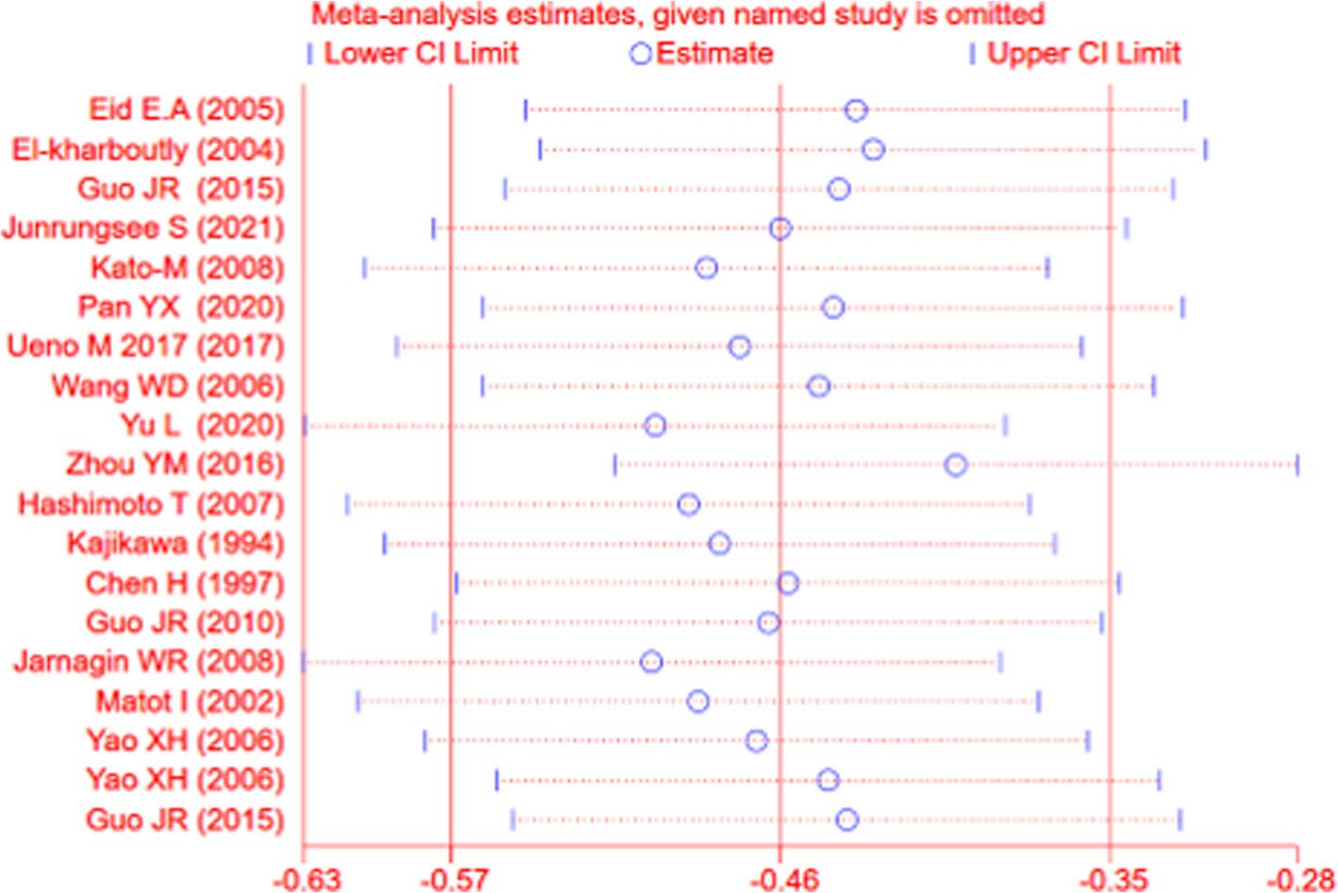
Sensitivity analysis for the blood loss amount

The sensitivity analysis showed that the estimated effect remained stable and within a limited range (Fig. 8).

#### GRADE evaluation

We assessed the quality of outcomes by GRADE evaluation and summarized in Table 3. Much of the evidence was judged as moderate certainty due to serious risk of bias. The *I*^*2*^ was high for the results of blood loss, blood transfusion volume and ALT, AST and BUN levels; therefore, GRADE evaluations were low (Table 3).

**Table 3:** Results of GRADE evaluation

**Outcomes**	**No of Participants** **(studies)**	**Quality of the evidence** **(GRADE)**
**Operative blood loss**	1236 (17 studies)	⊕ ⊕ ⊝ ⊝ **Low**
**Number requiring blood transfusion**	810 (12 studies)	⊕ ⊕ ⊕ ⊝ **moderate**
**Total blood transfusion**	180 (5 studies)	⊕ ⊕ ⊝ ⊝ **low**
**Perioperative complications**	793 (8 studies)	⊕ ⊕ ⊕ ⊝ **moderate**
**Operating time**	941 (12 studies)	⊕ ⊕ ⊕ ⊝ **moderate**
**Mortality**	760 (9 studies)	⊕ ⊕ ⊕ ⊝ **moderate**
**ALT—POD1**	241 (3 studies)	⊕ ⊕ ⊝ ⊝ **low**
**ALT—POD3**	151 (2 studies)	⊕ ⊕ ⊝ ⊝ **low**
**ALT—POD7**	241 (3 studies)	⊕ ⊕ ⊝ ⊝ **low**
**AST—POD1**	191 (2 studies)	⊕ ⊕ ⊝ ⊝ **low**
**AST—POD3**	180 (2 studies)	⊕ ⊕ ⊕ ⊝ **moderate**
**AST—POD7**	191 (2 studies)	⊕ ⊕ ⊕ ⊝ **moderate**
**TBIL—POD1**	241 (3 studies)	⊕ ⊕ ⊕ ⊝ **moderate**
**TBIL—POD3**	230 (3 studies)	⊕ ⊕ ⊕ ⊝ **moderate**
**TBIL—POD7**	241 (3 studies)	⊕ ⊕ ⊕ ⊝ **moderate**
**BUN—POD1**	236 (3 studies)	⊕ ⊕ ⊝ ⊝ **low**
**BUN—POD3**	236 (3 studies)	⊕ ⊕ ⊝ ⊝ **low**
**BUN—POD7**	151 (2 studies)	⊕ ⊕ ⊕ ⊝ **moderate**
**Cr—POD1**	404 (5 studies)	⊕ ⊕ ⊕ ⊝ **moderate**
**Cr—POD3**	314 (4 studies)	⊕ ⊕ ⊕ ⊝ **moderate**
**Cr—POD7**	319 (4 studies)	⊕ ⊕ ⊝ ⊝ **low**
**Hospital stay**	356 (3 studies)	⊕ ⊕ ⊕ ⊝ **moderate**

*Abbreviations: GRADE* Grading of Recommendations, Assessment, Development, and Evaluation, *ALT* Alanine transaminase, *AST* Aspartate aminotransferase, *TBIL* Total bilirubin, *BUN* Blood urea nitrogen, *CR* Creatinine

## Discussion

This meta-analysis included 17 trials with a total of 1,296 patients and evaluated the safety and efficacy of different cardiovascular interventions during hepatectomy. Our analysis revealed that the CVP-lowering strategy effectively decreased the blood loss amount and transfusion requirements, which is consistent with previous findings. Moreover, LCVP with IVC clamping reduced the incidence of postoperative complications. Blood dilution and ANH reduced the number of patients requiring allogeneic blood transfusions, although they did not reduce intraoperative bleeding. There were no significant differences in the mortality rate or postoperative liver and kidney functions between the cardiovascular intervention and control groups.

It is well known that massive blood loss and transfusion negatively impact perioperative outcomes [36]. Undoubtedly, blood transfusion is essential in improving tissue perfusion and oxygenation but increases the risk of transfusion-transmitted infection and is associated with increased tumour recurrence secondary to immunosuppressive effects [37, 38]. Cardiovascular interventions aim to reduce intra- and postoperative blood losses and allogeneic blood transfusions; however, their safety and efficacy in patients undergoing hepatectomy remain controversial.

In hepatectomy, a CVP of < 5 mmHg is considered low. Different measures can be applied to maintain a CVP of < 5 mmHg during hepatectomy and are mainly divided into two categories: anaesthetic techniques and surgical methods. Anaesthetic techniques include Trendelenburg positioning, anaesthetic drug or vasodilator administration, and intravenous fluid infusion restriction. However, these techniques cannot always reduce the CVP to < 5 mmHg. Clamping the infrahepatic IVC might also reduce the CVP during transection of the liver parenchyma [39].

Previous reviews [4, 13, 15] have evaluated the effect of a low CVP on operative bleeding and found its advantages in reducing blood loss and blood transfusion requirements compared with those of a normal CVP during liver surgery, which provided guidance for clinical practice. Although a CVP-lowering strategy has been gradually accepted as a standard intervention recently, controversy remains regarding whether the CVP is a reliable parameter for intravascular volume assessment and regarding the effectiveness of CVP reduction, as several trials have reported that a low CVP is not directly associated with reduced blood loss [40, 41].

Our review included the latest published RCTs and strived to comprehensively include more studies irrespective of the publication language and country, especially those that applied the IVC clamping technique because of the controversy regarding the safety of this technique. In our review, the CVP was < 5 mmHg in the patients who received the anaesthetic technique and/or IVC clamping and > 5 mmHg in the controls. A reduced CVP may decrease blood flow resistance in the hepatic vein and sinusoids, thereby decreasing operative retrograde bleeding during hepatectomy [8]. Similar to previous reviews of RCTs, our review showed that a CVP-lowering strategy is associated with reduced blood loss and allogeneic blood transfusion requirements. Reducing bleeding and/or effective haemostasis provides a clean surgical field and shortens the operating time, which are beneficial for patients.

Nevertheless, the results for blood loss were heterogeneous. A number of factors might explain this great variation, including the type of surgery, presence of other active interventions, sample size, and year of publication. Additionally, the methodology of the techniques used to reduce the CVP should also be considered when assessing outcomes. The subgroup analysis comparing IVC clamping to achieve a controlled low CVP with no IVC clamping showed that the patients who received IVC clamping had a smaller blood loss amount and less requirement for blood transfusion than those who did not. Meanwhile, the six remaining trials that used the anaesthetic technique showed similar results.

Although LCVP has been widely regarded as standard practice during liver surgery, there are still concerns regarding its potential to reduce perfusion to important organs [15]. A major criticism of LCVP is based on the fact that intentional reduction of venous return inevitably leads to a lack of abdominal organ perfusion, particularly in the liver and kidneys. Moreover, prolonged controlled hypotension aggravates organ ischemia and hypoperfusion. Such complications could negate the benefits of having a low CVP and small blood loss amount during surgery [42]. Therefore, we also focused on the impact of a low CVP on postoperative morbidity and mortality. We found no differences in the liver and kidney functions between the patients with low and normal CVPs; the liver and kidney functions of the low CVP group returned to baseline even more rapid than did those of the control group. Interestingly, the incidence of adverse events after surgery declined in the low CVP group compared with that in the control group, which is different from the findings of previous meta-analysis [14, 15]. Nearly all trials reported improved postoperative recovery (significantly reduced rates of complications) in the low CVP group, while only Pan et al. [20] reported that venous gas embolism occurred slightly more frequently (but not significant) in the controlled LCVP group.

We believe that the decreased rate of postoperative complications is likely attributed to the improved techniques for lowering CVP. The percentage of studies that applied IVC clamping in our meta-analysis was higher than that in previous ones [14, 15]. Traditional anaesthetic techniques, such as intravenous fluid restriction, vasodilation, and anaesthetic drug administration, help to maintain a state of hypovolaemia and vasodilation and reduce the hepatic venous pressure, thus reducing venous bleeding during hepatic transection. IVC clamping potentially lowers the hepatic venous pressure without the requirement of fluid restriction, which might protect patients from microcirculatory disturbances due to hypovolaemia and improve the outcomes. However, IVC clamping has been reported to be related to a higher risk of deep vein thrombosis and pulmonary embolism [43]. Therefore, we performed a subgroup analysis of postoperative morbidity. The forest plots showed that the patients who received IVC clamping had a reduced complication rate compared with those who did not; meanwhile, a similar rate of morbidity was found for the anaesthetic technique between the two groups. Hughes et al. [14] also compared the complication rates between IVC and non-IVC clamping groups and found no intergroup differences. A possible explanation is that the CVP was < 5 mmHg not only in the low CVP group but also in the control group. Therefore, it is unsurprising that no different outcomes were observed in that review. Our findings further support the importance of a well-controlled low CVP; however, the optimal CVP requires further investigation.

Autologous blood donation is recommended to avoid allogeneic blood transfusions. Herein, we found that autologous blood donation did not reduce blood loss but greatly decreased the number of patients requiring allogeneic blood transfusions. The two trials that evaluated the effect of autologous blood donation showed that 22% of patients in the control group received allogeneic blood transfusion compared with 8% of patients in autologous blood donation group. This not only reduces the risk of allogeneic transfusion-transmitted disease, but also avoid transfusion-related immunomodulatory effects associated with allogeneic transfusion, thereby reducing hospital stay and postoperative complication rates and relieving the financial burden on patients [44]. In addition to the risk of allogeneic transfusion-transmitted disease, allogeneic transfusion may also have long-term effect on immunity, leading to microthrombosis, blood coagulation, and hemolytic reactions, thereby prolonging the hospital stay and increasing the economic burden of patients and their families. Autologous blood donation can avoid the serious harm caused by allogeneic transfusion and alleviate the problem of blood shortage. However, with advanced public health measures, rigorous pre-donor screening, and donor blood testing, there has been a sharp decline in the risk of transfusion-transmitted infection. Simultaneously, concerns on the adverse effects associated with autologous blood donation have gradually increased [45]. The two trials did not report systemic complications, so the present meta-analysis failed to estimate this outcome. Therefore, the clinical benefit of autologous blood donation is not unequivocally supported by evidence.

Intraoperative ANH is another blood conservation technique with the advantages of lower cost and less inconvenience to patients. In our study, ANH did not significantly reduce operative blood loss but reduced red blood cell loss as a result of blood dilution, although the volume was the same. Generally, the reinfusion of autologous blood can reduce the volume of allogeneic blood transfused or even avoid allogeneic blood transfusion. Theoretically, patients should (1) have a relatively high haematocrit level; (2) undergo the maximum permissible number of phlebotomy; and (3) have a massive blood loss amount of > 1 L or 20% of the blood volume during surgery to maximise the advantage of ANH in reducing bleeding and transfusion requirements [46]. To date, high-quality evidence in favour of the routine use of ANH remains lacking. The number of eligible trials was small, and participants enrolled in some trials were too few (< 10 patients in each group), leading to a low statistical power and likely biased results [47]. Four trials reported the occurrence of adverse events, such as mortality and postoperative complications, and no differences were found between ANH and control treatment.

A potential explanation for the unexpected result in the morbidity rates for ANH is the small number of participants in the included RCTs, which may not be sufficient to detect differences in postoperative complications. Meanwhile, the explanation for the similar incidence of postoperative complications and mortality is the potentially detrimental effect of ANH. For example, low Hb concentrations often occur in patients receiving ANH, and artificial anaemia reduces the oxygen-carrying capacity of the blood, leading to tissue hypoxia.

Although this meta-analysis did not show that the reduction in the transfusion requirements by ANH and autologous blood donation ultimately leads to a significantly reduced rate of perioperative outcomes, the advantages of these interventions aimed at reducing perioperative bleeding and transfusion requirements in patients undergoing liver transection still have practical implications. Additionally, the different anaesthesia techniques, surgical approaches, and surgeons’ assessments of the ease of surgery are important factors in improving short- and long-term outcomes, which is worthy of further exploration in the future. Recently, with the evolution of surgical techniques, surgeries associated with large blood loss amounts no longer require transfusions; however, whether ANH is still beneficial for patients undergoing hepatectomy remains unclear [48]. Perhaps a combination of a part or all blood management techniques used for suitable patients in certain circumstances would become the new practice.

Herein, we also included two trials that simultaneously applied two interventions. The two trials evaluated the utilisation of LCVP combined with hypotension control and low CVP combined with ANH. The benefit appears to be greater with the combination of two interventions.

The main limitation of this review was the heterogeneity of the primary outcomes owing to the various surgical approaches and LCVP techniques used. The impact of the methodology used to reduce the CVP on the outcomes could not be underestimated. Another potential reason for the heterogeneity could be the long-time span of the included studies, which might have led to the application of different surgical and anaesthetic approaches. Furthermore, the inclusion of only a few trials for each intervention and the small sample size might have led to a high risk of types I and II errors. Therefore, trials with larger sample sizes are warranted. Lastly, the low quality of the included studies owing to unclear allocation concealment, selective reporting, and the lack of blinding methods was also a principal limitation.

## Conclusion

In summary, this meta-analysis demonstrates that LCVP can effectively reduce blood loss and improve clinical outcomes and that all cardiovascular interventions can reduce blood transfusion requirements during hepatectomy. The effectiveness and safety of LCVP, especially in combination with IVC clamping, are further confirmed in adult patients undergoing hepatectomy. Thus, the application of this strategy should be supported. ANH and autologous blood donation as a part of blood management should be used for suitable patients in certain circumstances. Prospective randomised trials with a low risk of systematic and random errors are needed to confirm the effects of these cardiovascular interventions in patients undergoing hepatectomy to optimise outcomes after surgery.

## Supplementary Information

**Additional file 1.** Search Strategy.

**Additional file 2: Supplementary Fig. 1. **Forest plot of the meta-analysis for the incidence of different types of complications. **Supplementary Fig. 2. **Forest plot of the meta-analysis for the operating time. **Supplementary Fig. 3.** Forest plot of the meta-analysis for the perioperative mortality rate. **Supplementary Fig. 4.** Forest plot of the meta-analysis for the ALT level. ALT, alanine transaminase. **Supplementary Fig. 5.** Forest plot of the meta-analysis for the AST level. AST, aspartate aminotransferase. **Supplementary Fig. 6. **Forest plot of the meta-analysis for the total bilirubin level. **Supplementary Fig. 7.** Forest plot of the meta-analysis for the BUN level. BUN, blood urea nitrogen. **Supplementary Fig. 8.** Forest plot of the meta-analysis for the Cr level. Cr, creatinine. **Supplementary Fig. 9.** Forest plot of the meta-analysis for the postoperative hospital stay length.

## Data Availability

The datasets supporting the conclusions of this article are included within the article.

## Abbreviations

ERAS: Ehanced recovery after surgery
LCVP: Lowering the central venous pressure
ANH: Acute normovolemic hemodilution
PRISMA: Reporting Items for Systematic Review and Meta-Analyses
RCTs: Randomized clinical studies
SD: Standard deviation
RoB 2: The Revised Cochrane risk-of-bias tool for randomized trials
GRADE: The Grading of Recommendations Assessment, Development, and Evaluation
ALT: Alanine transaminase
AST: Aspartate aminotransferase
TB: Total bilirubin
BUN: Blood urea nitrogen
Cr: Serum creatinine
RRs: Risk ratios
CIs: Confidence intervals
MD: Mean difference
IQR: Interquartile range
IVC: Inferior vena cava
HCC: Hepatocellular carcinoma
PLC: Primary liver carcinoma
MLT: Metastatic liver tumor
GBC: Gallbladder carcinoma
ICC: Intrahepatic cholangiocellular carcinoma
MCC: Metastasis of colorectal carcinoma
