# Supplementary material for: Application of cardiovascular interventions to decrease blood loss during hepatectomy: a systematic review and meta-analysis
Source: BMC Anesthesiol. 2023 Mar 22;23:89. doi: 10.1186/s12871-023-02042-y (PMC10032024; doi:10.1186/s12871-023-02042-y)
Supplement: Supplementary file 1 — Additional file 1. Search Strategy. [file 12871_2023_2042_MOESM1_ESM.docx]

**Search Strategy**

**PubMed:**

(Blood loss OR bleeding OR hemorrhage OR haemorrhage OR hemorrhages OR haemorrhages OR hemostasis OR haemostasis OR transfusion OR "Hemorrhage"[Mesh] OR "Blood Transfusion"[Mesh]) AND (((liver OR hepatic OR hepato OR "liver"[Mesh]) AND (resection OR segmentectomy)) OR hepatectomy OR "hepatectomy"[Mesh]) AND (((randomized controlled trial [pt] OR controlled clinical trial [pt] OR randomized controlled trials [mh] OR random allocation [mh] OR clinical trial [pt] OR clinical trials [mh] OR ("clinical trial" [tw]) OR (placebos [mh] OR placebo* [tw] OR random* [tw] OR research design [mh:noexp]) NOT (animals [mh] NOT human [mh])))).

**Cochrane library**

(Blood loss OR bleeding OR hemorrhage OR hemostasis OR transfusion) AND (((liver OR hepatic OR hepato) AND (resection OR segmentectomy)) OR hepatectomy)

**Embase**

1 exp CROSSOVER PROCEDURE/

2 exp DOUBLE BLIND PROCEDURE/

3 exp SINGLE BLIND PROCEDURE/

4 exp RANDOMIZED CONTROLLED TRIAL/

5 (((RANDOM* or FACTORIAL* or CROSSOVER* or CROSS) and OVER*) or PLACEBO* or ASSIGN* or ALLOCAT* or VOLUNTEER*).af.

6 1 or 2 or 3 or 4 or 5

7 exp BLEEDING/

8 exp Blood Transfusion/

9 (Blood loss or bleeding or hemorrhage or hemostasis or transfusion).af.

10 8 or 7 or 9

11 (liver or hepatic or hepato).af.

12 (segmentectomy or resection).af.

13 11 and 12

14 hepatectomy.af.

15 exp Liver Resection/

16 13 or 15 or 14

17 6 and 16 and 10

**Web of Science**

#1 TS=(Blood loss OR bleeding OR hemorrhage OR hemostasis OR transfusion)

#2 TS=(((liver OR hepatic OR hepato) AND (resection OR segmentectomy)) OR hepatectomy)

#3 TS=(random* OR blind* OR placebo* OR meta-analysis)

#4 #3 AND #2 AND #1
