# Supplementary figures and images for: Application of cardiovascular interventions to decrease blood loss during hepatectomy: a systematic review and meta-analysis
Source: BMC Anesthesiol. 2023 Mar 22;23:89. doi: 10.1186/s12871-023-02042-y (PMC10032024; doi:10.1186/s12871-023-02042-y)

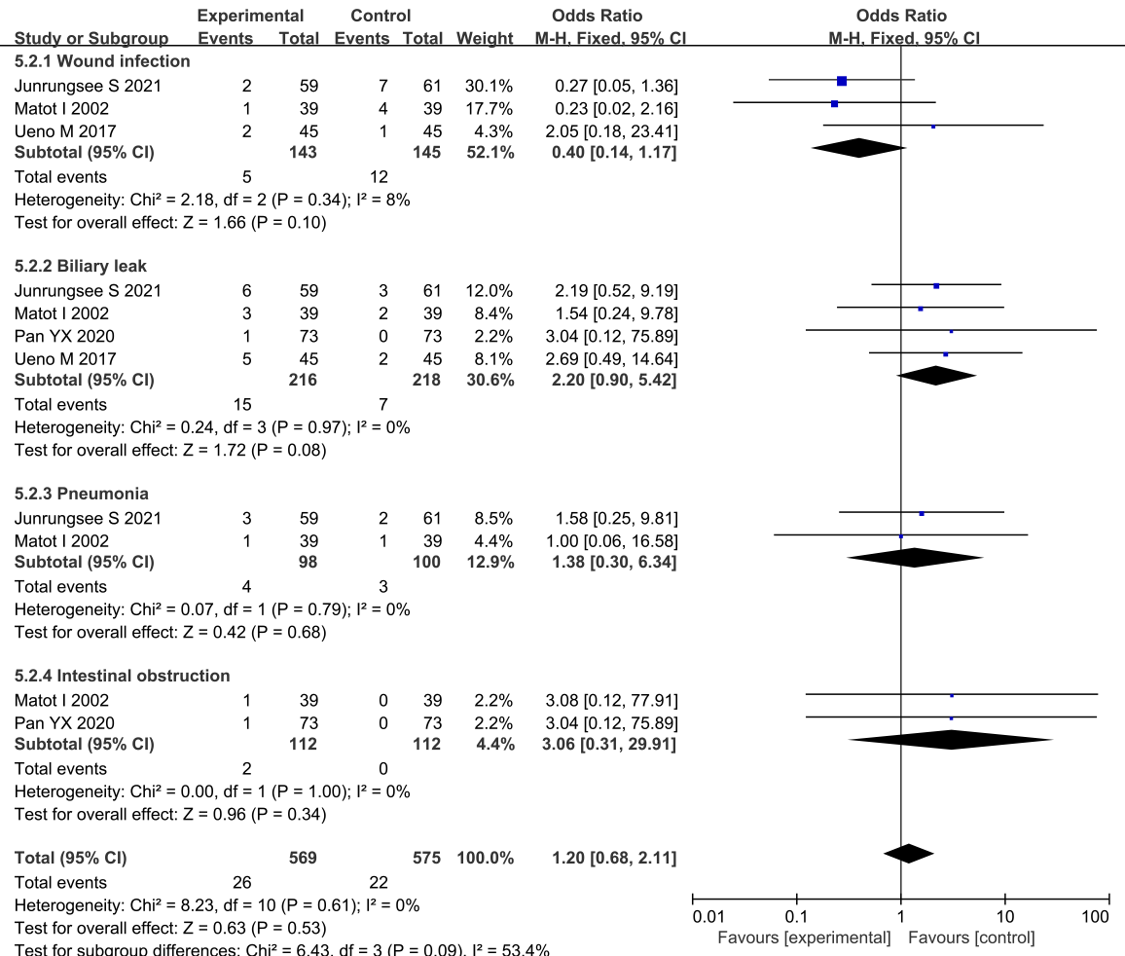

Supplement: Supplementary file 2 — Additional file 2: Supplementary Fig. 1. Forest plot of the meta-analysis for the incidence of different types of complications. Supplementary Fig. 2. Forest plot of the meta-analysis for the operating time. Supplementary Fig. 3. Forest plot of the meta-analysis for the perioperative mortality rate. Supplementary Fig. 4. Forest plot of the meta-analysis for the ALT level. ALT, alanine transaminase. Supplementary Fig. 5. Forest plot of the meta-analysis for the AST level. AST, aspartate aminotransferase. Supplementary Fig. 6. Forest plot of the meta-analysis for the total bilirubin level. Supplementary Fig. 7. Forest plot of the meta-analysis for the BUN level. BUN, blood urea nitrogen. Supplementary Fig. 8. Forest plot of the meta-analysis for the Cr level. Cr, creatinine. Supplementary Fig. 9. Forest plot of the meta-analysis for the postoperative hospital stay length. [file 12871_2023_2042_MOESM2_ESM.zip › Supplementary figure 1.tif]

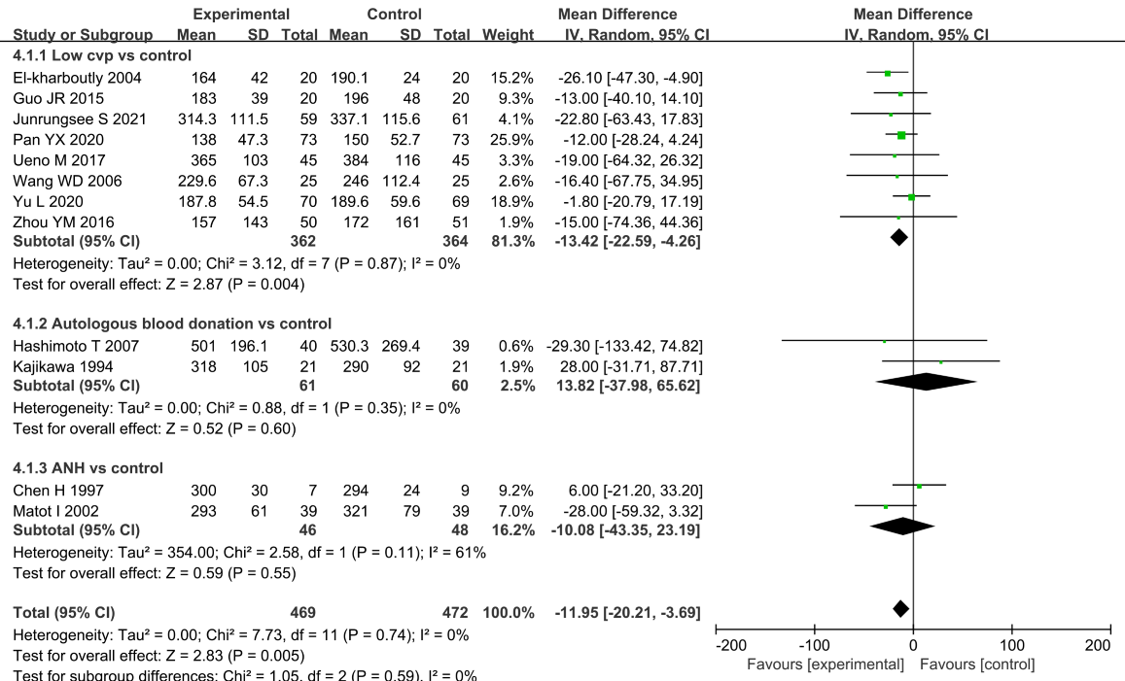

Supplement: Supplementary file 2 — Additional file 2: Supplementary Fig. 1. Forest plot of the meta-analysis for the incidence of different types of complications. Supplementary Fig. 2. Forest plot of the meta-analysis for the operating time. Supplementary Fig. 3. Forest plot of the meta-analysis for the perioperative mortality rate. Supplementary Fig. 4. Forest plot of the meta-analysis for the ALT level. ALT, alanine transaminase. Supplementary Fig. 5. Forest plot of the meta-analysis for the AST level. AST, aspartate aminotransferase. Supplementary Fig. 6. Forest plot of the meta-analysis for the total bilirubin level. Supplementary Fig. 7. Forest plot of the meta-analysis for the BUN level. BUN, blood urea nitrogen. Supplementary Fig. 8. Forest plot of the meta-analysis for the Cr level. Cr, creatinine. Supplementary Fig. 9. Forest plot of the meta-analysis for the postoperative hospital stay length. [file 12871_2023_2042_MOESM2_ESM.zip › Supplementary figure 2.tif]

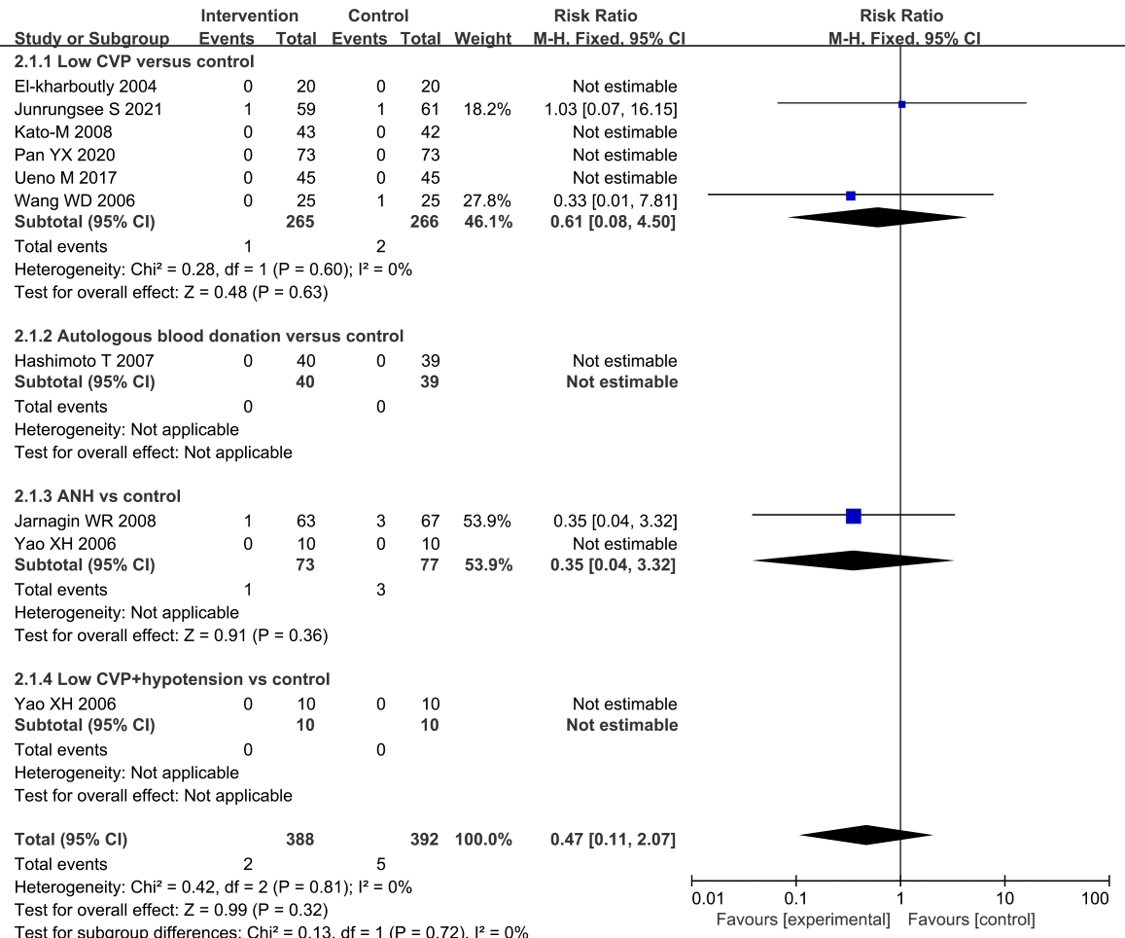

Supplement: Supplementary file 2 — Additional file 2: Supplementary Fig. 1. Forest plot of the meta-analysis for the incidence of different types of complications. Supplementary Fig. 2. Forest plot of the meta-analysis for the operating time. Supplementary Fig. 3. Forest plot of the meta-analysis for the perioperative mortality rate. Supplementary Fig. 4. Forest plot of the meta-analysis for the ALT level. ALT, alanine transaminase. Supplementary Fig. 5. Forest plot of the meta-analysis for the AST level. AST, aspartate aminotransferase. Supplementary Fig. 6. Forest plot of the meta-analysis for the total bilirubin level. Supplementary Fig. 7. Forest plot of the meta-analysis for the BUN level. BUN, blood urea nitrogen. Supplementary Fig. 8. Forest plot of the meta-analysis for the Cr level. Cr, creatinine. Supplementary Fig. 9. Forest plot of the meta-analysis for the postoperative hospital stay length. [file 12871_2023_2042_MOESM2_ESM.zip › Supplementary figure 3.tif]

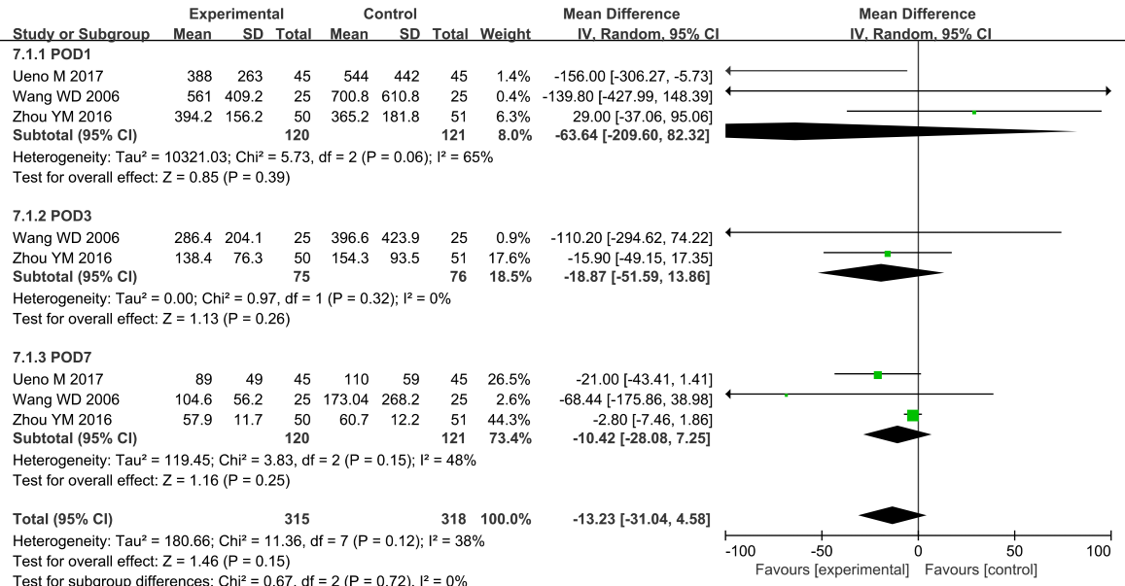

Supplement: Supplementary file 2 — Additional file 2: Supplementary Fig. 1. Forest plot of the meta-analysis for the incidence of different types of complications. Supplementary Fig. 2. Forest plot of the meta-analysis for the operating time. Supplementary Fig. 3. Forest plot of the meta-analysis for the perioperative mortality rate. Supplementary Fig. 4. Forest plot of the meta-analysis for the ALT level. ALT, alanine transaminase. Supplementary Fig. 5. Forest plot of the meta-analysis for the AST level. AST, aspartate aminotransferase. Supplementary Fig. 6. Forest plot of the meta-analysis for the total bilirubin level. Supplementary Fig. 7. Forest plot of the meta-analysis for the BUN level. BUN, blood urea nitrogen. Supplementary Fig. 8. Forest plot of the meta-analysis for the Cr level. Cr, creatinine. Supplementary Fig. 9. Forest plot of the meta-analysis for the postoperative hospital stay length. [file 12871_2023_2042_MOESM2_ESM.zip › Supplementary figure 4.tif]

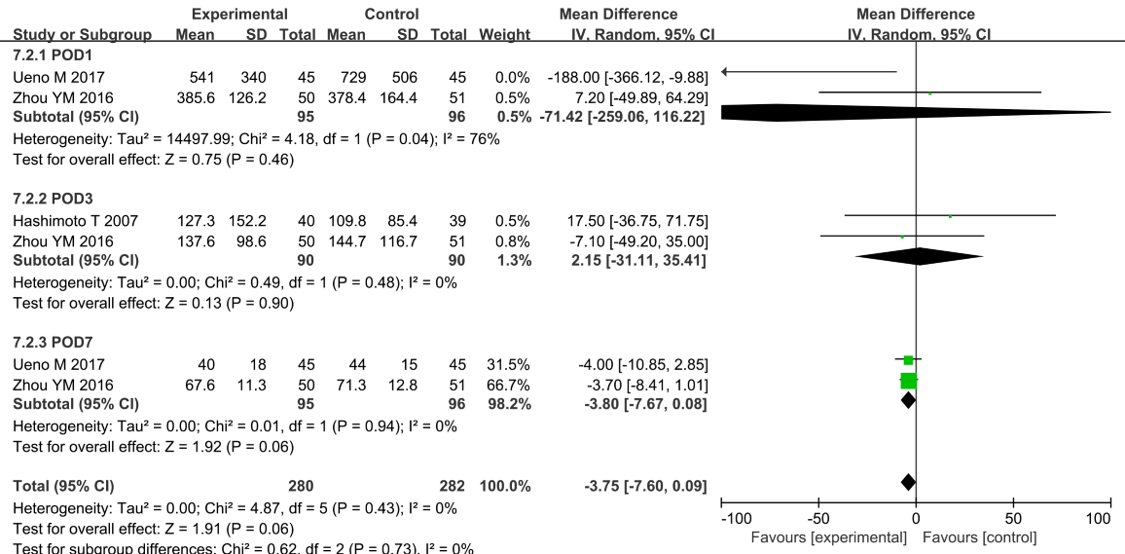

Supplement: Supplementary file 2 — Additional file 2: Supplementary Fig. 1. Forest plot of the meta-analysis for the incidence of different types of complications. Supplementary Fig. 2. Forest plot of the meta-analysis for the operating time. Supplementary Fig. 3. Forest plot of the meta-analysis for the perioperative mortality rate. Supplementary Fig. 4. Forest plot of the meta-analysis for the ALT level. ALT, alanine transaminase. Supplementary Fig. 5. Forest plot of the meta-analysis for the AST level. AST, aspartate aminotransferase. Supplementary Fig. 6. Forest plot of the meta-analysis for the total bilirubin level. Supplementary Fig. 7. Forest plot of the meta-analysis for the BUN level. BUN, blood urea nitrogen. Supplementary Fig. 8. Forest plot of the meta-analysis for the Cr level. Cr, creatinine. Supplementary Fig. 9. Forest plot of the meta-analysis for the postoperative hospital stay length. [file 12871_2023_2042_MOESM2_ESM.zip › Supplementary figure 5.tif]

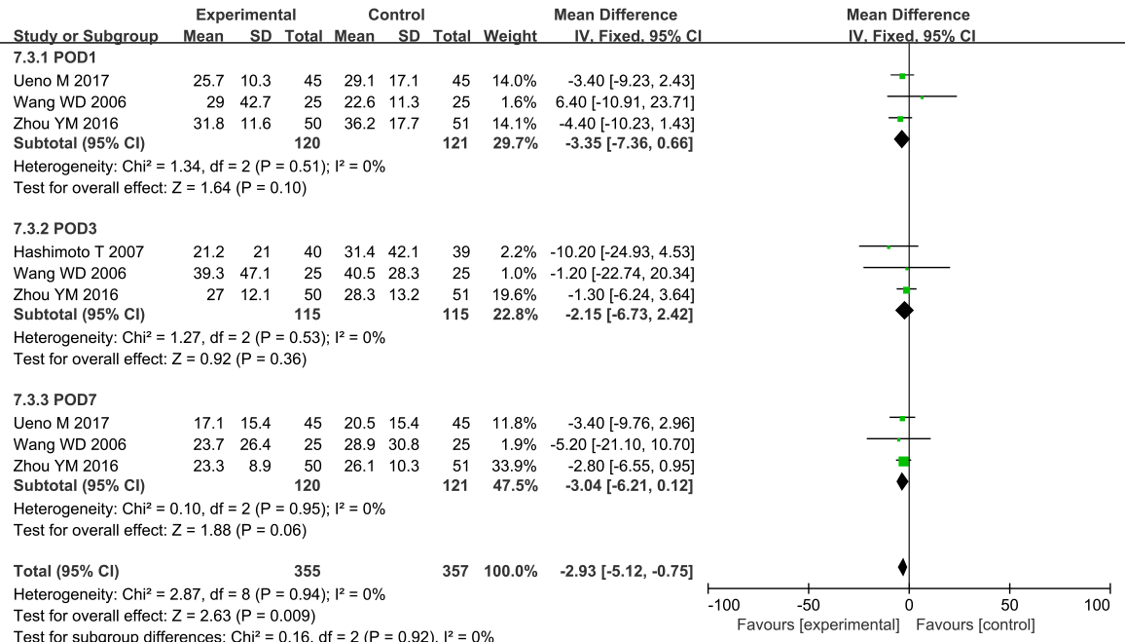

Supplement: Supplementary file 2 — Additional file 2: Supplementary Fig. 1. Forest plot of the meta-analysis for the incidence of different types of complications. Supplementary Fig. 2. Forest plot of the meta-analysis for the operating time. Supplementary Fig. 3. Forest plot of the meta-analysis for the perioperative mortality rate. Supplementary Fig. 4. Forest plot of the meta-analysis for the ALT level. ALT, alanine transaminase. Supplementary Fig. 5. Forest plot of the meta-analysis for the AST level. AST, aspartate aminotransferase. Supplementary Fig. 6. Forest plot of the meta-analysis for the total bilirubin level. Supplementary Fig. 7. Forest plot of the meta-analysis for the BUN level. BUN, blood urea nitrogen. Supplementary Fig. 8. Forest plot of the meta-analysis for the Cr level. Cr, creatinine. Supplementary Fig. 9. Forest plot of the meta-analysis for the postoperative hospital stay length. [file 12871_2023_2042_MOESM2_ESM.zip › Supplementary figure 6.tif]

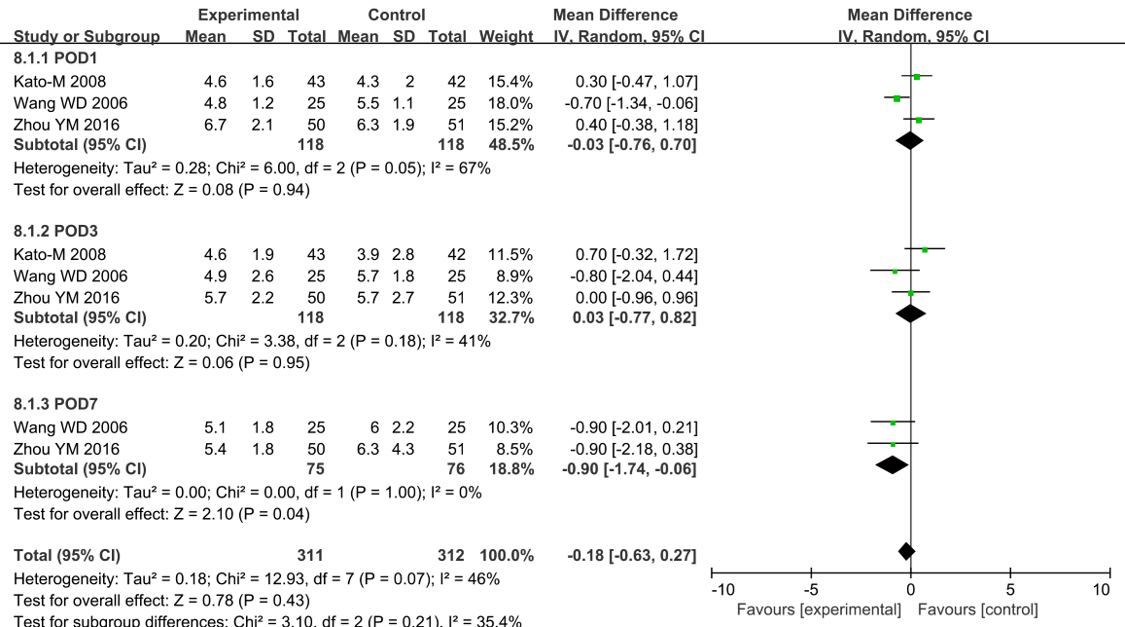

Supplement: Supplementary file 2 — Additional file 2: Supplementary Fig. 1. Forest plot of the meta-analysis for the incidence of different types of complications. Supplementary Fig. 2. Forest plot of the meta-analysis for the operating time. Supplementary Fig. 3. Forest plot of the meta-analysis for the perioperative mortality rate. Supplementary Fig. 4. Forest plot of the meta-analysis for the ALT level. ALT, alanine transaminase. Supplementary Fig. 5. Forest plot of the meta-analysis for the AST level. AST, aspartate aminotransferase. Supplementary Fig. 6. Forest plot of the meta-analysis for the total bilirubin level. Supplementary Fig. 7. Forest plot of the meta-analysis for the BUN level. BUN, blood urea nitrogen. Supplementary Fig. 8. Forest plot of the meta-analysis for the Cr level. Cr, creatinine. Supplementary Fig. 9. Forest plot of the meta-analysis for the postoperative hospital stay length. [file 12871_2023_2042_MOESM2_ESM.zip › Supplementary figure 7.tif]

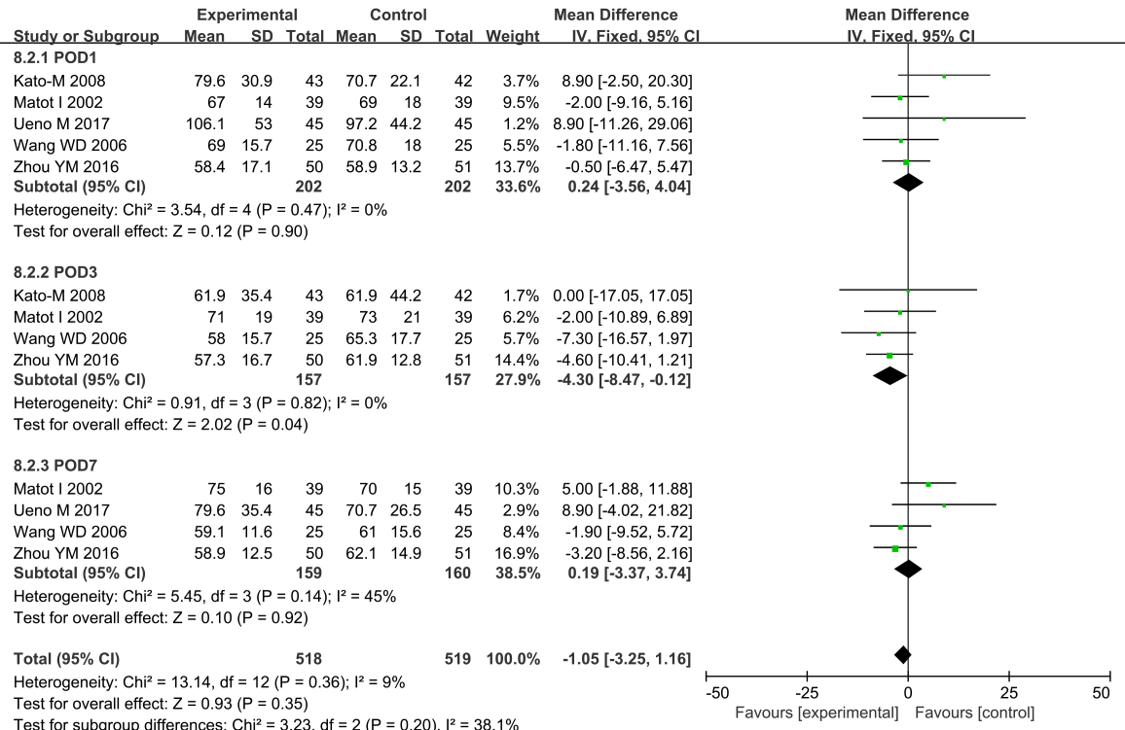

Supplement: Supplementary file 2 — Additional file 2: Supplementary Fig. 1. Forest plot of the meta-analysis for the incidence of different types of complications. Supplementary Fig. 2. Forest plot of the meta-analysis for the operating time. Supplementary Fig. 3. Forest plot of the meta-analysis for the perioperative mortality rate. Supplementary Fig. 4. Forest plot of the meta-analysis for the ALT level. ALT, alanine transaminase. Supplementary Fig. 5. Forest plot of the meta-analysis for the AST level. AST, aspartate aminotransferase. Supplementary Fig. 6. Forest plot of the meta-analysis for the total bilirubin level. Supplementary Fig. 7. Forest plot of the meta-analysis for the BUN level. BUN, blood urea nitrogen. Supplementary Fig. 8. Forest plot of the meta-analysis for the Cr level. Cr, creatinine. Supplementary Fig. 9. Forest plot of the meta-analysis for the postoperative hospital stay length. [file 12871_2023_2042_MOESM2_ESM.zip › Supplementary figure 8.tif]

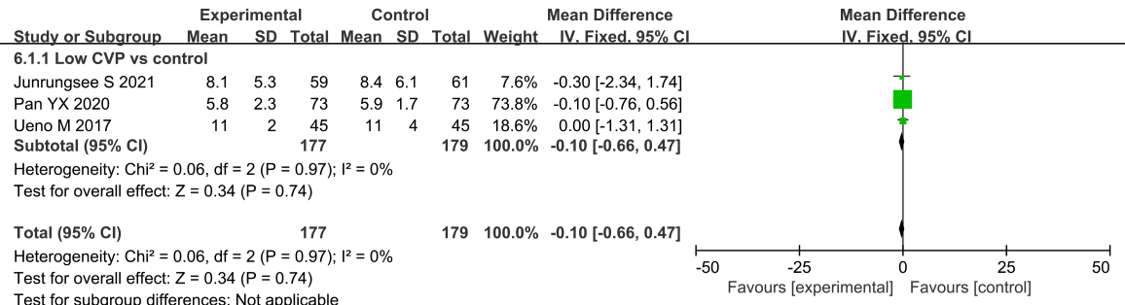

Supplement: Supplementary file 2 — Additional file 2: Supplementary Fig. 1. Forest plot of the meta-analysis for the incidence of different types of complications. Supplementary Fig. 2. Forest plot of the meta-analysis for the operating time. Supplementary Fig. 3. Forest plot of the meta-analysis for the perioperative mortality rate. Supplementary Fig. 4. Forest plot of the meta-analysis for the ALT level. ALT, alanine transaminase. Supplementary Fig. 5. Forest plot of the meta-analysis for the AST level. AST, aspartate aminotransferase. Supplementary Fig. 6. Forest plot of the meta-analysis for the total bilirubin level. Supplementary Fig. 7. Forest plot of the meta-analysis for the BUN level. BUN, blood urea nitrogen. Supplementary Fig. 8. Forest plot of the meta-analysis for the Cr level. Cr, creatinine. Supplementary Fig. 9. Forest plot of the meta-analysis for the postoperative hospital stay length. [file 12871_2023_2042_MOESM2_ESM.zip › Supplementary figure 9.tif]
